# The content of hippocampal “replay”

**DOI:** 10.1002/hipo.22824

**Published:** 2018-01-10

**Authors:** Brad E. Pfeiffer

**Affiliations:** ^1^ Department of Neuroscience UT Southwestern Medical Center Dallas Texas 75390

**Keywords:** memory, place cells, reactivation, review, ripple

## Abstract

One of the most striking features of the hippocampal network is its ability to self‐generate neuronal sequences representing temporally compressed, spatially coherent paths. These brief events, often termed “replay” in the scientific literature, are largely confined to non‐exploratory states such as sleep or quiet rest. Early studies examining the content of replay noted a strong correlation between the encoded spatial information and the animal's prior behavior; thus, replay was initially hypothesized to play a role in memory formation and/or systems‐level consolidation via “off‐line” reactivation of previous experiences. However, recent findings indicate that replay may also serve as a memory retrieval mechanism to guide future behavior or may be an incidental reflection of pre‐existing network assemblies. Here, I will review what is known regarding the content of replay events and their correlation with past and future actions, and I will discuss how this knowledge might inform or constrain models which seek to explain the circuit‐level mechanisms underlying these events and their role in mnemonic processes.

## 1 INTRODUCTION

Adaptive behavior requires the brain to preserve coherent representations of experience and later extract that stored information to inform future behaviors. For decades, researchers have utilized goal‐directed navigation and the brain's spatial memory system as a specific example to explore more general questions regarding memory processes (Tolman, 1948). Two discoveries in particular have prompted researchers to focus such studies on the hippocampus: (a) Human patients and animal models with damage to their medial temporal lobe (and the hippocampus in particular) display severe impairments in their ability to form new episodic and spatial memories (Morris et al., 1982; Scoville & Milner, 1957; Squire et al., 2004); and (b) individual neurons within the hippocampus represent spatial information in their firing patterns during exploration, implicating the hippocampus in the processing and storage of spatial memories (Moser et al., 2008; O'Keefe and Dostrovsky, 1971). A persistent, fundamental question within this domain is how an entire experience or spatial trajectory, which may be represented within the hippocampus by the activity of tens of thousands of neurons ordered in a precise temporal sequence, can be coherently stored within that neural network or purposefully retrieved at the exact time when the stored information would be useful for guiding future decisions.

**Figure 1:**
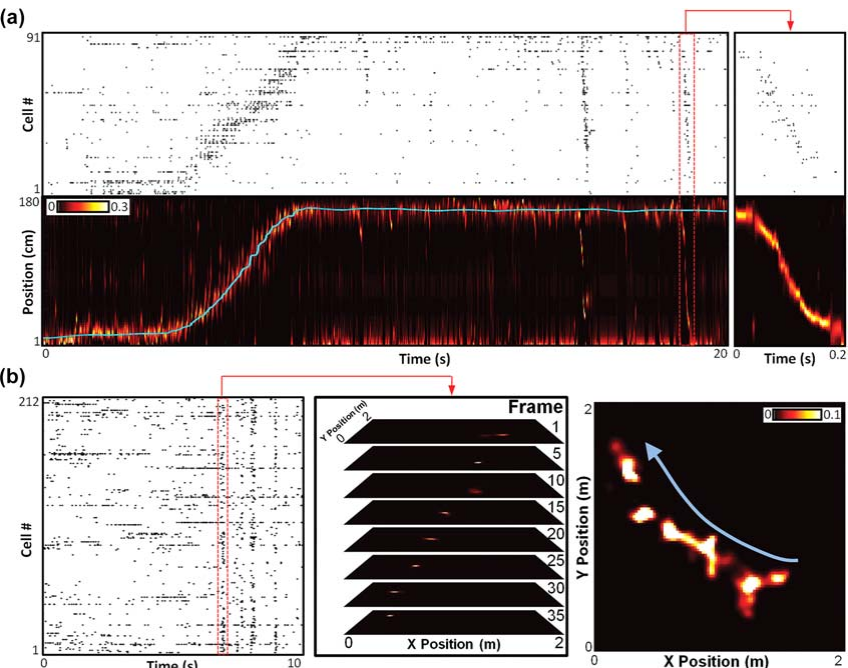
Examples of hippocampal replay. (a) Example of a linear track replay. Top: Raster plot of 91 simultaneously monitored CA1 place cells recorded while a rat traversed a 1.8‐m‐long linear track, ordered by location of place field peak. Bottom: Cyan line is the rat's actual position along the track during the recording in the top panel. Background color map indicates the rat's estimated position probabilities based on Bayesian decoding of the spike trains in the top panel (20 ms decoding window, advanced in 5 ms increments). Red‐bordered section indicates the spikes and estimated position for one ripple, showing a replay of a trajectory across the track, expanded on the right. (b) Example of an open field replay. Left: Raster plot of 212 simultaneously monitored CA1 place cells recorded while a rat explored a 2 m × 2 m open arena. Middle: Bayesian estimated position probabilities in selected frames from the highlighted ripple‐based spikes. Right: Sum of all decoded frames of the highlighted ripple, demonstrating an encoded trajectory crossing the center of the open arena in the direction of the blue arrow. Data from Pfeiffer and Foster (2015)

Among several lines of investigation attempting to address this question (Garner et al., 2012; Josselyn et al., 2015; Ramirez et al., 2013; Tse et al., 2007), hippocampal ripples and ripple‐based “replay” have emerged as strong candidate mechanisms which may facilitate both the initial storage and later retrieval of complex, temporally ordered information about experience. Ripples are brief (50–100 ms duration), high‐frequency (150–300 Hz) network oscillations within the hippocampus which emerge during non‐exploratory states such as slow‐wave sleep, quiet rest, grooming, and eating/drinking (Buzsáki, 1986, 2015), and disruption in their expression results in significant memory impairments (Ego‐Stengel & Wilson, 2010; Girardeau et al., 2009; Jadhav et al., 2012; Nakashiba et al., 2009; Nokia et al., 2012; Wang et al., 2015a). Ripples are the physiological signature of coherent, temporally structured population‐level events within the hippocampus in which the pattern of neural activity often appears to encode behaviorally relevant information on a compressed timescale (Davidson et al., 2009; Foster & Wilson, 2006; Lee & Wilson, 2002) (Figure 1). Intriguingly, the content of ripple‐based activity patterns can range between forwards‐ordered or reverse‐ordered replay of prior behaviors (Ambrose et al., 2016; Diba & Buzsáki, 2007; Foster & Wilson, 2006; Karlsson & Frank, 2009), prediction of future actions (Dragoi & Tonegawa, 2011; Pfeiffer & Foster, 2013; Singer et al., 2013; Ólafsdóttir et al., 2015; Wu et al., 2017), or even assimilation of independent experiences into a novel creation (Gupta et al., 2010; Pfeiffer & Foster, 2013). Thus, terms commonly used to describe this phenomenon, such as “replay” or “reactivation,” fail to adequately capture the diversity of function which these events likely represent. For simplicity and consistency with prior literature, however, I will use the term “replay” to describe all content‐expressing ripple‐based hippocampal sequences.

Recently, several excellent reviews have been published which explore the brain's spatial representation network (Moser et al., 2015) and hippocampal ripples (Buzsáki, 2015). Here, I will attempt to minimize overlap with these articles and instead focus this review on the information content within hippocampal replay and its relationship to prior and future behavior with the premise that a more thorough understanding of *what* is encoded by ripples may provide insight into questions regarding *how* and ultimately *why* replay is expressed.

## 2 EARLY STUDIES INDICATE THAT SLEEP REPLAY REPLICATES PRIOR NEURONAL ACTIVITY PATTERNS

It is important to place our current understanding of replay in context by first examining a brief history of hippocampal reactivation studies. Interpretations of early results were heavily influenced by prominent theoretical models which described a two‐stage process for memory formation (Buzsáki, 1989; Marr, 1971). Briefly, the models proposed that initial experience produces a labile and likely transient memory trace in the hippocampal network via activity‐dependent plastic changes to the circuit. During subsequent “off‐line” states (e.g., sleep), repeated ripple‐based reactivation of precise hippocampal neural sequences associated with prior behavior was hypothesized to either consolidate those plastic changes within the hippocampus or transfer the information to the slower‐adapting cortical circuit for long‐term storage. These models and their subsequent iterations (Buzsáki, 1996, 1998; O'Neill et al., 2010; Redish & Touretzky, 1998) were attractive for several reasons: (a) they provided an explanation for anterograde amnesia and temporally graded retrograde amnesia following hippocampal damage (Scoville & Milner, 1957; Squire et al., 2004); (b) they brought together emerging discoveries in hippocampal plasticity and *in vivo* physiology to explain sequentially ordered memory (Bliss & Lomo, 1973; Buzsáki et al., 1983; Buzsaki (1986); Zhang et al., 1998; Dan & Poo, 2004); and (c) they beautifully addressed the temporal credit assignment problem (Schultz et al., 1997) by proposing that hippocampal reactivation would allow for the temporal compression of prior experience to bring action and outcome together within the same brief circuit‐level activity pattern.

In perhaps the earliest report of hippocampal reactivation supporting the two‐stage model, Pavlides and Winson established that individual hippocampal neurons which were highly active during spatial exploration displayed an increase in their overall activity during subsequent sleep compared to control neurons whose activity had not been elevated during the behavioral session (Pavlides & Winson, 1989). In a subsequent landmark study, Wilson and McNaughton provided the first evidence for multi‐neuron reactivation by demonstrating that pairs of hippocampal neurons which reliably fired within 100 ms of one another during exploration were also likely to fire together during sleep sessions after, but not before, the behavior (Wilson & McNaughton, 1994). Importantly, it was noted that the post‐experience pair‐wise correlations were significantly stronger during ripples than at other times during sleep (Wilson & McNaughton, 1994), providing the first experimental evidence that ripples may serve as an electrophysiological marker for off‐line reactivation. Subsequent reports demonstrated that the precise temporal order of firing during waking behavior was preserved in sleep‐based ripples at both the level of neuron pairs (Hirase et al., 2001; Kudrimoti et al., 1999; Shen et al., 1998; Skaggs & McNaughton, 1996) as well as larger, multi‐neuron ensembles (Ji & Wilson, 2007; Lee & Wilson, 2002; Nádasdy et al., 1999). In addition, the studies on ensemble reactivation consistently reported that sleep‐based sequences were temporally compressed when compared to their corresponding waking sequences (Ji & Wilson, 2007; Lee & Wilson, 2002; Nádasdy et al., 1999). Thus, early studies on hippocampal reactivation indicated that prior behaviors may be re‐expressed during sleep‐based ripples on a compressed timescale, generating support and considerable excitement for the two‐stage model of memory formation and establishing the term “replay” in the scientific literature. Regarding the mechanisms underlying replay expression, these initial findings strongly supported the hypothesis that patterns of neuronal activity occurring during behavior could strengthen the activated synapses via Hebbian plasticity (Dan & Poo, 2004; Feldman, 2012; Hebb, 1949), and these modified synaptic weights would then serve as a framework for the eventual re‐expression of those same patterns during ripples, effectively allowing the flow of neural activity to follow the synaptic “path of least resistance”. Indeed, the rate of sequential reactivation in post‐experience sleep was initially reported to be dependent upon the rate of activity correlation during prior behavior, supporting the notion that Hebbian processes underlie replay expression (Jackson et al., 2006; O'Neill et al., 2006, 2008).

## 3 AWAKE REPLAY PROVIDES UNEXPECTED INSIGHTS

While considerable early work focused on sleep‐based reactivation, it was known that ripples occur not only in slow‐wave sleep, but also during brief pauses in exploratory activity during the awake state (Buzsáki et al., 1983), and initial pairwise correlation studies indicated that awake ripples may also replay experience‐relevant sequences (Jackson et al., 2006; Kudrimoti et al., 1999; O'Neill et al., 2006). In a groundbreaking study, Foster and Wilson reported that awake replay did not always conform to the established pattern (Foster & Wilson, 2006). Rather, awake reactivation, while still a temporal compression of prior activity, could progress in the opposite temporal sequence to that which had initially occurred during behavior, as if the prior neural activity patterns were being replayed in reverse (Foster & Wilson, 2006). Many subsequent studies replicated this surprising finding (Csicsvari et al., 2007; Davidson et al., 2009; Diba & Buzsáki, 2007; Gupta et al., 2010; Karlsson & Frank, 2009; Wu & Foster, 2014), and the term “reverse replay” entered the lexicon. Presciently, reverse reactivation of prior activity in ripples had been predicted to arise from the rekindling of recently stimulated synaptic traces nearly two decades earlier in one of the first models of two‐stage memory formation (Buzsáki, 1989). Importantly, many studies also demonstrated “forward replay”—reactivation of prior sequences in the original order in which they occurred—encoded by ripples in the awake state (Davidson et al., 2009; Diba & Buzsáki, 2007; Gupta et al., 2010; Wu & Foster, 2014), further complicating the picture of awake replay.

Many of the initial studies on awake replay utilized relatively simple environments, such as linear or two‐choice tracks. However, despite (or perhaps because of) the limited behavioral repertoires observed in these experiments, several critical observations were made regarding the content of replay which both inform and constrain models of replay expression.

### 3.1 Replay requires minimal experience

Several groups have shown that experience and experience‐dependent synaptic plasticity are correlated with and/or required for robust replay expression (Dupret et al., 2010; Jackson et al., 2006; Silva et al., 2015; but see Section 3.2). However, reverse replay can arise following only a single traversal across a novel track (Foster & Wilson, 2006; Gupta et al., 2010), demonstrating that behavioral repetition is not strictly necessary for subsequent re‐expression of neural activity patterns and further indicating that plastic changes arising from a single behavioral trial are sufficient to modify the hippocampal circuit to allow for subsequent replay expression.

Notably, theta‐phase‐ordered spiking of neurons during exploration (O'Keefe & Recce, 1993) produces temporally compressed sequences of neuronal activity known as “theta sequences” (Dragoi & Buzsáki, 2006; Foster & Wilson, 2007; Johnson & Redish, 2007; Skaggs et al., 1996), which are repeated multiple times during a single epoch of movement. Pairwise activity during theta sequences occurs on a timescale that is appropriate for spike‐timing dependent plasticity (STDP) (Dan & Poo, 2004; Feldman, 2012), suggesting that theta sequences may facilitate long‐lasting changes to the underlying circuit which allow for the subsequent re‐expression of those same sequences in ripples. In an important test of this hypothesis, reproduction of recorded *in vivo* activity patterns of hippocampal place cell pairs in *ex vivo* hippocampal slices reliably produced long‐term potentiation, but only when the place fields of the recorded neurons overlapped (Isaac et al., 2009). Furthermore, cholinergic signaling, which increases in the hippocampus during active exploration and is linked to theta sequence expression (Colgin, 2013; Douchamps et al., 2013; Wang et al., 2015b), was required to induce plasticity (Isaac et al., 2009).

**Figure 2:**
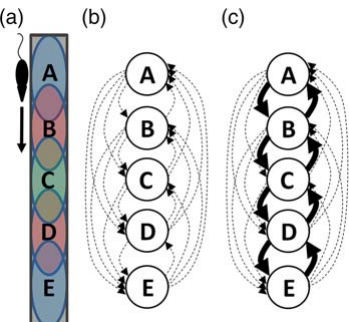
Simple model of reverse replay of direction‐specific place cells. The discovery of symmetric STDP rules in hippocampal area CA3 (Mishra et al., 2016) supports the following simple model of reverse replay. (a) Layout of population‐level CA3 place fields along a linear track. Colored ovals indicate place fields of large neural populations. For example, neural population A is composed of dozens to hundreds of CA3 neurons which all have place fields at the top of the track, while population E is composed of a distinct set of neurons which all have place fields at the bottom of the track. Due to place field directionality (McNaughton et al., 1983; Muller et al., 1994), these particular populations only fire when the rat is running from the top to the bottom of the track and are silent when the rat runs in the reverse direction. Thus, the population sequence during running is always A→B→C→D→E (the sequence E→D→C→B→A never occurs during behavior). (b) Simple network connectivity model. Assuming random initial connectivity, some neurons in population A form excitatory synapses on other neurons in population A (not shown), while some form synapses on neurons in the other populations (arrows). (c) Following a single lap across the track, symmetric STDP between neurons with overlapping fields results in strengthening of synapses in both directions (represented by thicker arrows). Subsequently when the rat is standing in place field E during a ripple, population E is likely to fire first given the current sensory input to the hippocampal circuit. Due to the established plasticity, the sequence E→D→C→B→A can emerge in the ripple despite never occurring during behavior

Complicating this explanation, it was recently demonstrated that theta sequences themselves are poorly organized on the first lap across a novel track (Feng et al., 2015), raising questions regarding their suitability for establishing replay after only one experience. STDP has been shown to be sensitive to even subtle perturbations in the timing of neural activity (Seol et al., 2007), indicating that the inconsistent temporal structure of early‐experience theta sequences may not be well‐suited for inducing plasticity. However, two pieces of evidence suggest that even poorly ordered theta sequences may support plasticity within the hippocampus. First, the presence of neuromodulators can regulate the polarity of STDP (Seol et al., 2007), suggesting that novelty or salience signals may facilitate synapse strengthening even when the precise timing of neural activity is unreliable. Second, STDP within the recurrent synapses of the hippocampal CA3 network display symmetric potentiation regardless of pre‐ versus post‐synaptic activity order (Mishra et al., 2016). Critically, symmetric STDP within a densely interconnected region of the hippocampus helps resolve the long‐standing question of how a temporally ordered pattern of activity across the circuit can be re‐expressed in the opposite temporal order, particularly for direction‐specific place fields (McNaughton et al., 1983; Muller et al., 1994; Figure 2). It is worth noting that the above studies examining synaptic plasticity utilized induction protocols lasting for several minutes (Isaac et al., 2009; Mishra et al., 2016; Seol et al., 2007), whereas reverse replay can occur after a single epoch of movement lasting only a few seconds (Foster & Wilson, 2006). Recent work suggests that such brief activity patterns are indeed capable of inducing long‐lasting potentiation through both Hebbian (Huang & Kandel, 2005; Redondo et al., 2010; Sajikumar et al., 2008; Villers et al., 2012) and non‐Hebbian mechanisms (Bittner et al., 2017).

### 3.2 Replay can encode temporally and spatially remote experiences

While it has been consistently demonstrated that there is a bias for the content of awake reactivation events to encode paths starting from the animal's current location and crossing the immediate environment (Csicsvari et al., 2007; Davidson et al., 2009; Karlsson & Frank, 2009; O'Neill et al., 2006; Pfeiffer & Foster, 2013; Wu & Foster, 2014), Karlsson & Frank reported that during brief pauses in exploration of one environment, replay could encode coherent trajectories in a physically separate, previously explored environment (Karlsson & Frank, 2009). The presence of spatially remote replay provides strong evidence that immediate sensory input is not the exclusive driver of replay content, even though external input can bias the content of replay (Bendor & Wilson, 2012; McNamara et al., 2014). Importantly, the existence of remote replay argues against models of replay as simple sequential activation of sub‐threshold place fields following circuit disinhibition (Foster and Wilson, 2006).

Early models of hippocampal function suggested that reverse replay may arise from reactivation of a slowly decaying activity trace (Buzsáki, 1989), which necessarily places a limitation on the temporal window in which reverse replay should be observable. However, Gupta et al. reported the presence of coherent replays up to 10 min after exploration of the represented environment (Gupta et al., 2010), raising questions regarding the suitability of the synaptic trace model.

Thus, these studies strongly argue that while the content of replay may be biased by current inputs, the internal circuitry of the hippocampus is capable of producing coherent replications of both spatially and temporally remote experiences (Gupta et al., 2010; Karlsson & Frank, 2009). Furthermore, because these data demonstrate that replay can represent previously learned information (in the form of spatially coherent and meaningful sequences) in the near‐complete absence of the sensory cues associated with the represented experiences, these studies provide strong evidence that replay represents a form of spatial memory expression.

### 3.3 Reward influences the content of replay

Replay has been postulated to solve the temporal credit assignment problem by combining the neuronal representations of both current outcome and prior behavior within a single, brief window of time (Cichosz, 1999; Foster & Wilson, 2006). A natural prediction of this hypothesis is that salience should play an important role in the quantity and/or content of replay. Indeed, several studies have found that representation of novel versus familiar environments is elevated in replay (Kudrimoti et al., 1999; McNamara et al., 2014; O'Neill et al., 2008). In addition, the rate of ripples was significantly increased at rewarding versus non‐rewarding sites (Singer & Frank, 2009). Many features of ripples, including the likelihood of cell participation, were also enhanced during reward‐based ripples, and these changes were even stronger when novel behavioral contingencies had to be learned (Singer & Frank, 2009).

In a subsequent study, Ambrose et al. studied the effect of reward on the information content of replay and replicated the correlation between total ripple rate and reward level (Ambrose et al., 2016). Importantly, however, the authors also found that the number of reverse‐ordered, but not forwards‐ordered, replays was significantly correlated to the magnitude of the reward (Ambrose et al., 2016), consistent with a model in which reverse replay may participate in memory consolidation of salient experiences, while forward replay may serve as a memory retrieval system for planning future behaviors (discussed in Section 4).

These discoveries further suggest that salience signals, such as reward‐ or novelty‐induced dopamine release, may serve to initiate the expression of replay and/or bias the content of replay. In a direct confirmation of this prediction, it was recently reported that optogenetic activation of dopaminergic inputs to the hippocampus enhanced the likelihood of ripple‐based pairwise reactivation reflecting a recently explored environment (McNamara et al., 2014). The underlying mechanisms through which neuromodulatory input can impact the rate and content of replay remain unclear (Miyawaki et al., 2014; Rosen et al., 2015).

### 3.4 Replay exhibits learned information

A fundamental question regarding replay is whether the encoded information content is a direct replication of actual experience or a more complicated representation of learned spatial relationships. Because the majority of early studies on replay utilized relatively simple environments with stereotyped behaviors, such as repeated traversals along linear tracks, it was challenging to detangle these two possibilities. In one of the first studies to record from sufficient numbers of neurons to accurately decode the information content within a given replay, Davidson et al. demonstrated that the virtual trajectories encoded by awake replay appeared to progress at constant velocities (roughly 15–20 times faster than the animal's average movement), even when the animal's behavior was irregular (Davidson et al., 2009), suggesting that ripples may express learned information rather than actual experience.

Powerful evidence in support of this notion came from experiments examining replay in rats exploring a two‐choice maze (Gupta et al., 2010). Despite the fact that the rats always traveled from the central arm to either the left or right arm during behavior, Gupta et al. observed replay events encoding a “shortcut” path from the left arm to the right arm, a path the rat had never actually taken (Gupta et al., 2010), demonstrating mental assembly of distinct experiences into a coherent representation of the spatial relationships within the environment. Similar mental construction was revealed in a subsequent study which reported unique combinations of forward‐ and reverse‐replay at choice points of a three‐arm maze (Wu & Foster, 2014).

### 3.5 Related experiences can be combined in replay via ripple concatenation

In the same study described above, replays were examined as rats explored a ten‐meter‐long track (Davidson et al., 2009). Importantly, because the virtual velocity within replays was relatively constant, playback of a ten‐meter trajectory required over half a second to complete, much longer than a typical 100 ms ripple (Buzsáki, 1986, 2015). The authors observed while individual ripples themselves appear to be somewhat restricted in their temporal duration, replay of long spatial trajectories can be represented by concatenating multiple ripples together, a finding which has been replicated by several groups (Davidson et al., 2009; Wu & Foster, 2014; Yamamoto & Tonegawa, 2017).

A question arising from these data is how does a ripple in the middle of an extended replay “know” where the last ripple ended so it can continue the encoded path in a coherent manner? A potential answer comes from a recent study in which hippocampal and entorhinal cortical activity was simultaneously monitored (Yamamoto & Tonegawa, 2017). During replay, the medial entorhinal cortex (MEC), which communicates bi‐directionally with the hippocampus (Witter et al., 2000), can also display increases in ripple‐band power (Roth et al., 2016; Yamamoto & Tonegawa, 2017). Indeed, replay has recently been reported to occur across populations of MEC grid cells (Ólafsdóttir et al., 2016; O'Neill et al., 2017), although caveats have been raised regarding the interpretation of grid cell sequences (Trimper et al., 2017). Intriguingly, awake ripple‐like events in MEC temporally alternated with concatenated ripples in the hippocampus, indicative of a recurrent loop of activity and further suggesting that the end representation of one hippocampal ripple within a longer chain may initiate a corresponding representation in the MEC, which can then send that information back to the hippocampus to allow the next concatenated ripple to start in that location. Consistent with this interpretation, inhibition of MEC activity reduced the number of multi‐ripple events without altering total ripple number and resulted in fragmented spatial representations within replay events (Yamamoto & Tonegawa, 2017).

If true, this process may underlie the fascinating feature of multi‐ripple replay in which a single extended replay will intersperse both forwards‐ and reverse‐ordered movement on consecutive ripples (Davidson et al., 2009; Wu & Foster, 2014): the “re‐ignition” of a chained ripple via MEC feedback may contain both location and momentum information, but the direction‐specific population that is activated may be stochastically determined by the local hippocampal network rather than extra‐hippocampal sources (Brandon et al., 2012). It should be noted, however, that while replay in the deep layers of MEC (which receive hippocampal output) may participate in hippocampal replay (Ólafsdóttir et al., 2016), a prior study found that replay in the superficial MEC (which provides input to the hippocampus) was not coincident with hippocampal replay (O'Neill et al., 2017), so the question of MEC involvement with replay is not fully resolved.

The chaining of multiple ripples may serve a more important function than simply allowing for representation of prolonged paths, as each ripple within the larger sequence appears to facilitate the combination of distinct spatial paths in physically plausible (but possibly novel) ways, perhaps to promote the construction of a mental model of the environment (Davidson et al., 2009; Gupta et al., 2010; Wu & Foster, 2014). Like reverse replays, joint replays comprised of ripple chains can arise from minimal experience (Wu & Foster, 2014).

## 4 FUTURE REPRESENTATION IN REPLAY— “REPLAY” IS NOT JUST REPLAY

A complicated picture of replay emerges in which ripples encode for sequences of learned information rather than simple replications of prior neuronal activity. In particular, an important functional distinction appears to exist between the information content of reverse‐ordered replay and forwards‐ordered replay. By replicating prior activity patterns starting from the present and extending into the animal's past, reverse replay seems ideally suited for memory consolidation of previous salient experience, linking result to prior action (Foster & Wilson, 2006). In contrast, forward replay in the awake state seems more practically suited for utilizing previously formed memories to sample possible future outcomes. Indeed, several lines of evidence support this latter hypothesis.

The first indication that awake forward replay may serve to construct predictions regarding future actions came from observations that this form of memory expression tends to arise immediately prior to movement (Ambrose et al., 2016; Diba & Buzsáki, 2007), suggestive of a planning or preparatory mechanism. Furthermore, replay appears capable of constructing novel shortcuts in which the specific paths encoded had never previously been experienced (Gupta et al., 2010), consistent with a hippocampal role in imagining future possibilities (Buckner, 2010). Dupret et al. also demonstrated that ripples occurring throughout a goal‐directed navigational task tend to represent learned goal locations (Dupret et al., 2010), although it was unclear from this study whether the observed replays were retrospective for past behavior or predictive of future behavior. Finally, blockade of ripples during awake behavior impairs performance on a spatial working memory task, consistent with a role of replay in memory retrieval (Jadhav et al., 2012).

### 4.1 Forward replay can correlate with future behavior

Singer et al. provided the first clear evidence that the content of replay can correspond to future actions (Singer et al., 2013). On a spatial alternation task in a two‐choice maze, they observed that following learning (when the animal's behavior was >85% correct), the content of forward replay immediately prior to making the spatial decision was significantly more likely to encode the correct path than the incorrect path. These data suggest that expression of a virtual trajectory in forward replay can influence the animal's future behavior.

This work was supported by similar findings in rats performing a goal‐directed navigational task in an open arena (Pfeiffer & Foster, 2013). In this study, rats alternated between two behaviors in the same familiar environment: random foraging and goal‐directed navigation to a recently learned location to obtain predictable reward. Importantly, the predictable goal changed location daily and the task was structured such that goal‐directed navigation to this newly learned position entailed unique combinations of start and end points throughout most of an experimental session. When the rat was away from the recently learned goal location, replay was strongly biased to encode spatial trajectories that started at the rat's current location and ended at the goal (Pfeiffer & Foster, 2013), indicating that replay is capable of rapidly assimilating prior knowledge (the spatial layout of the arena) with newly learned information (the current goal location) in a way that can inform behavior. Indeed, during goal‐directed navigation, the rat's future behavioral trajectories were strongly correlated with the paths encoded by replay events.

### 4.2 Forward replay can encode paths to avoid

Additional evidence that replay may provide a foundation for mental exploration of possible future actions comes from a study testing the role of replay in avoidance behavior (Wu et al., 2017). After initial exploration of a linear track, rats were given a pair of mild shocks when they reached one end of the track. During subsequent exploration of the track, rats displayed consistent avoidance of the shock zone, stopping and turning around before entering it. Replays during these pauses reliably encoded trajectories leading into the shock zone immediately prior to the rat turning around, consistent with a model of replay as a memory retrieval system capable of providing outcome predictions. Importantly, though, in this study the replay denoted paths to avoid rather than paths to follow, indicating that the content of replay is used to inform rather than dictate future behavior, possibly by coordinating the reactivation of amygdala‐based representations of the valence of the encoded experience (Girardeau et al., 2017).

Several important points should be noted regarding the above studies, which pose challenges to coherent models of hippocampal replay.

### 4.3 Reverse replay does not facilitate goal learning in a familiar environment

During open field navigation (Pfeiffer & Foster, 2013), replays that occurred after the rat arrived at the recently learned goal location did not display a bias to encode paths corresponding to its prior path, suggesting that reverse replay was not prominent in this task and may therefore be dispensable for the process of rapidly assigning novel salience to a familiar location. These data are at odds with a clear enhancement of reward‐based reverse replay in linear tracks (Ambrose et al., 2016; Singer & Frank, 2009). While this may be partly explained by the fact that the open field environment was highly familiar in the open field study, the predictable reward location was novel every session, and it is reasonable to expect strong release of salience signals at that position (Schultz et al., 1997). Instead, these data suggest that reverse replay may be primarily utilized in the initial formation of a mental map of a novel environment (Carr et al., 2011; Roux et al., 2017), serving to synaptically couple neurons that represent adjacent locations; once formed, other processes may flexibly assign value to specific positions within that cognitive map. A prediction from this hypothesis is that replay in a novel open arena should predominantly encode the rat's prior behavioral path, even if the rat is performing a familiar, goal‐directed task that was learned in a separate environment. It is important to note, however, that reverse replay is reliably observed during exploration of highly familiar tracks (Gupta et al., 2010), suggesting that it likely continues to serve additional purposes beyond initial memory formation.

### 4.4 Forward replay correlates to behavior only prior to memory‐driven action

The encoding of paths leading to salient locations and the strong correlation between the content of replay and the rat's future behavior was only observed prior to goal‐seeking (Pfeiffer & Foster, 2013) or active avoidance (Wu et al., 2017). In contrast, replays which occurred prior to simple exploration or random foraging were significantly less correlated to the animal's future behavior in these studies. At first glance, these data seem to indicate that in the absence of a predictable, salient outcome, the content of replay was not effective at influencing behavior, suggesting the presence of a top‐level “evaluator” that assesses the content and result of a forward replay and decides whether to utilize that information or not. However, Pfeiffer and Foster demonstrated that during goal‐seeking periods, when the content of replay (rarely) encoded paths leading somewhere other than the goal location, the rat was still highly likely to follow these paths even though they often took the rat farther away from the learned goal (Pfeiffer & Foster, 2013), arguing against the evaluator model (or at least, arguing against a perfect, pure‐reward‐based evaluator).

### 4.5 Future behavior is not strictly determined by forward replay

Finally, while the correlation between the content of replay and the rat's future behavior was quite strong prior to goal‐directed navigation in an open field (Pfeiffer & Foster, 2013), it was far from deterministic. Although many behaviors closely matched the entire path encoded by the previous replay, some followed different paths to the same endpoint, others followed the encoded trajectory for a short time and then diverged, while a few never overlapped at all. Thus, while these studies provide strong evidence that the content of replay is correlated with planned action, the precise relationship between forward replay and behavior is not trivially explained.

### 4.6 A simple model of goal‐directed forward replay

**Figure 3:**
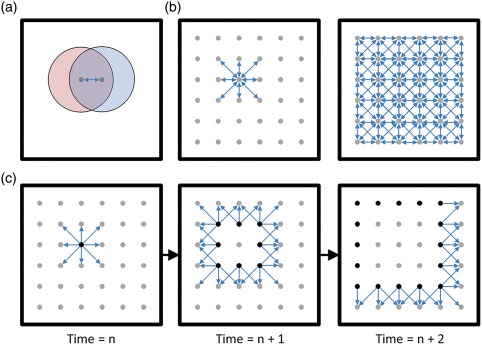
A simple feed‐forward model of replay fails to explain open field replay. (a) Top‐down view of a large open arena. Each dot represents a neural population with similar spatial representation. The place fields of two neuron populations are shown in red and blue. In this simple model, repeated traversal of the overlapping sections of these place fields serves to co‐activate these neuron populations sufficiently to strengthen the synaptic connections between them (represented with the bi‐directional arrow). (b) Left: Extended exploration of the environment would therefore serve to strengthen synaptic connections between populations of neurons representing adjacent locations. Right: This pattern of synaptic connectivity would be expected to exist across the entire cognitive map (assuming relatively equal exploration of all regions of the environment). (c) Using only simple feed‐forward network dynamics, the above model predicts that open field replay would encode a circular “wave” of representation that would start at the animal's current location (due to current sensory input driving those cells) and sweep in all directions simultaneously, following the established synaptic paths. Black dots indicate currently active neurons, arrows indicate currently active synapses. Critically, this is not what is observed *in vivo* (see Figure 1b), suggesting that more complicated network dynamics exist which regulate neuronal participation, likely involving inhibitory circuitry to force the representation to a single spatial location at a time

It is difficult to envision how a circuit can internally generate a goal‐directed path in an open arena with a novel combination of start and end location (Pfeiffer & Foster, 2013). Indeed, it is not immediately obvious that an open field replay would encode a path at all as opposed to a ring of spatial representation expanding from the animal's current location (Figure 3). Many models of replay presume that experience serves to establish an underlying map of connectivity, strengthening the connections between neurons with overlapping or adjacent place fields. In such a simple incarnation, an activity bump at the animal's current location during a ripple in an open field may be expected to activate all neurons with adjacent place fields, resulting in a circle of representation that would expand through the environment as neurons activate each of their spatial neighbors in turn. This clearly does not occur (Pfeiffer & Foster, 2013, 2015) and suggests more complex circuit‐level dynamics emphasizing lateral inhibition to encode precise, singular locations throughout the replay. It remains unclear, however, how a particular path to a known goal is expressed out of the near‐infinite number of possible paths.

While repeated rewarding traversals within an environment may serve to specifically strengthen the connections associated with those paths to allow for their subsequent re‐expression during replay, in the Pfeiffer & Foster study, the hippocampus effectively demonstrated zero‐trial prediction, encoding a specific path from a random starting location to a newly learned goal location—a path that had never previously been rewarded (Pfeiffer & Foster, 2013). This form of mental construction is not surprising given the known role of the hippocampus in future imagining (Buckner, 2010; Gaesser et al., 2013; Gupta et al., 2010), but the mechanisms which underlie this phenomenon are unknown. At least two models may account for this form of goal‐directed replay.

Sarel et al. recently reported that hippocampal neurons in bats can specifically encode the distance and direction to a goal (Sarel et al., 2017). One interpretation of these data is that the hippocampus may form two overlapping maps: one representing actual spatial relationships and the other representing the relative relationship between the animal and one or more salient locations. Upon exploration and discovery of a salient location, the spatial map and relational map can be aligned to provide distance/direction‐to‐goal information from any given spatial location. While computationally advantageous, the biological plausibility of this model remains to be determined.

**Figure 4:**
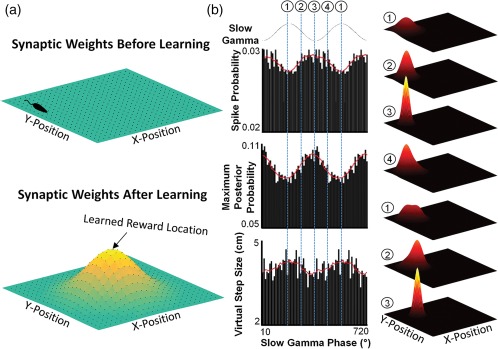
Model of goal‐directed open field replay. (a) Map of hippocampal connectivity weights in a square open arena before (top) and after (bottom) reward learning. Color map represents the strength of the synaptic connection between neural populations (dots) representing adjacent locations. Top: Following extended exploration prior to reward learning, neurons representing adjacent locations will be strongly connected to each other, with all connection strengths relatively equivalent (as denoted by the flat connectivity map). Bottom: Following repeated reward at a single site in the center of the arena, synaptic weights acquire a non‐uniform pattern, with stronger connections nearest the reward location. (b) Model of gamma‐based auto‐ and hetero‐associative dynamics underlying spatial trajectory representation during a ripple after reward learning. Left: Three measures of circuit‐level activity during ripples as a function of the slow gamma oscillation (Pfeiffer & Foster, 2015): spike probability (i.e., population firing rate), maximum Bayesian decoded posterior probability (i.e., the precision of the spatial representation within the hippocampus), and the virtual step size (i.e., the likelihood of the spatial representation in the hippocampus transitioning from one location to another). Circled numbers and dashed lines indicate distinct phases of the slow gamma oscillation. Right: Hippocampal spatial representation during a replay (heat map) across the indicated phases of the slow gamma oscillation. (1) Total spike count is low, resulting in a broad, non‐specific spatial representation. (2) As the gamma cycle progresses, spike rates increase. Via auto‐associative dynamics, place cells with overlapping place fields are preferentially activated, while lateral inhibition limits the representation of adjacent locations, tightening the spatial representation. (3) At the trough of the gamma cycle, firing rates are highest, resulting in the strongest auto‐association. Spatial representation is the most precise and accordingly the least likely to transition to a new location. (4) The decrease of excitatory drive on the ascending phase of gamma reduces overall firing rate, weakening the auto‐association and allowing hetero‐associative processes to begin to activate neural populations representing nearby locations. The neurons with the strongest synaptic connectivity to the initially active population are the most likely to be activated during this phase; thus, given the distribution of synaptic weights in the bottom of panel (a), the spatial representation will have the highest probability of transitioning toward the goal location. The cycle repeats several times, with auto‐associative dynamics dominating during periods of high firing rate to ensure high fidelity representation of individual locations and hetero‐associative dynamics dominating during periods of lower population activity in which the spatial representation moves according to established network synaptic weights (and thus moving toward locations of known salience)

Alternatively, the hippocampal map may not display equivalent connection strengths between all locations. Instead, neurons with overlapping fields nearer salient locations (such as predictable rewards), may develop stronger connections, resulting in a gradient of connection strengths across the environment (Figure 4). This pattern of asymmetrical synaptic weights may arise from a number of physiological origins, including enhanced synaptic plasticity due to increased release of a reward neuromodulator as the rat approaches the salient location (van der Meer & Redish, 2011), or a recently identified non‐Hebbian form of plasticity (Bittner et al., 2017). During a subsequent replay, the activity bump would likely initiate at the animal's current location, presumably due to immediate sensory inputs. It was previously shown that replay consists of alternating epochs of auto‐associative and hetero‐associative processes which serve to focus internal representation on a single location before moving to an adjacent location (Pfeiffer & Foster, 2015). During the hetero‐associative phase, the most likely neurons to be activated would be those with the strongest synaptic connections to the previously active population; thus, the non‐uniform distribution of synaptic weights across the network would “pull” the network representation toward areas of largest synaptic strength, and the highest probability path would lead toward the goal.

For simplicity, these models ignore the contribution of local inhibitory circuitry or extra‐hippocampal inputs in determining the content of replay. However, it is clear that cortical input can bias the content of replay (Bendor & Wilson, 2012; Jadhav et al., 2015; Yamamoto & Tonegawa, 2017), and a complete model of hippocampal replay will likely involve multiple cell types and interconnected brain areas.

## 5 REPLAY MAY REVEAL THE UNDERLYING HIPPOCAMPAL CIRCUITRY

While current evidence indicates that ripples can be influenced by external inputs (Bendor & Wilson, 2012; McNamara et al., 2014), these population‐level events are largely dependent upon the internal circuitry of the hippocampus (Buzsáki, 2015; Buzsáki et al., 1987). Thus, a fundamental assumption regarding ripple expression is that the content of replay should reflect some aspect of the underlying patterns and strengths of the hippocampal connectome. Further, it is reasonable to expect that those same functional connection patterns may influence how the hippocampal circuit processes information during active exploration. A hypothesis arising from these assumptions is that the sequential order of neuronal activity during ripples prior to a novel experience may correlate to the order in which those neurons will represent subsequent behavior.

In a series of high‐profile studies, Dragoi & Tonegawa directly tested these assumptions (Dragoi & Tonegawa, 2011, 2013a). Reminiscent of the earliest studies on replay (Kudrimoti et al., 1999; Wilson & McNaughton, 1994), hippocampal activity was recorded before, during, and after animal subjects explored a novel linear track. Surprisingly, neural activity during many pre‐experience ripples appeared to be sequentially organized to reflect the order that the cells would eventually fire during subsequent exploration of the never‐before‐seen track (Dragoi & Tonegawa, 2011, 2013a), as if the hippocampus was “pre‐playing” spatial paths that it would take in the future. Based on these data, the authors argued that the hippocampal map is, at least in part, pre‐configured. It is important to note that the authors did not rule out the possibility that experience‐dependent changes to the hippocampal circuit could serve to further strengthen or differentiate spatial representations (Dragoi & Tonegawa, 2013b).

These results remain controversial, as they challenge many established tenets. In particular, evidence from prior studies has indicated that experience is critical for observing coherent replay (Foster & Wilson, 2006; Jackson et al., 2006; Kudrimoti et al., 1999; O'Neill et al., 2008; Wilson & McNaughton, 1994). Indeed, subsequent work has explicitly tested the role of experience‐dependent synaptic plasticity in replay expression and the authors reported no evidence of pre‐play (Silva et al., 2015). It is possible that differences in statistical methods account for the discrepancy between pre‐play observation across different groups (Foster, 2017).

A potential resolution to this controversy was recently presented by Grosmark and Buzsáki (2016). In this study, the authors identified two functionally distinct populations of hippocampal neurons displaying either rigid or plastic network properties during replay. Rigid cells displayed higher overall firing rates with less spatially precise activity, possibly representing a highly connected, pre‐configured circuit. Plastic cells, on the other hand, displayed fewer, more precise place fields, presumably serving to distinguish independent environments. Importantly, while rigid cells had strong representation in replay events before and after exploration of a novel environment, participation of plastic cells was heavily weighted to post‐experience replay. Together, these data indicate that experience may serve to bind plastic cells into a background network structure provided by rigid cells. Given recent findings that hippocampal map may not be as stable as once thought (Attardo et al., 2015; Rubin et al., 2015; Ziv et al., 2013), the ability to flexibly add neurons to an existing framework may facilitate, rather than hinder, rapid acquisition of information (Tse et al., 2007), or it may serve as a mechanism allowing for generalization (Xu & Sudhof, 2013).

## 6 CONCLUSIONS

The above summary of replay content paints a complicated picture of this phenomenon. What was once considered a straightforward reflection of prior activity patterns instead encompasses a diverse array of content. Reverse replay, biased strongly by novelty and reward, seems well‐suited for modifying the circuitry of the hippocampus to consolidate learned spatial relationships, while forward replay appears capable of retrieving these stored representations to influence future behavior. Yet despite considerable work on the circuit‐level mechanisms underlying ripple generation (Buzsáki, 2015), it remains unclear how specific paths are selected for expression. Importantly, there is no current evidence indicating that forward and reverse replays are generated via distinct network mechanisms; therefore, identifying how the hippocampal circuitry selects for the expression of forward versus reverse replay (Ambrose et al., 2016) may provide clues as to how the same circuitry can represent specific paths during open field replay (Pfeiffer & Foster, 2013). Sleep and wake replay also seem fundamentally different, as blocking sleep‐based replays impairs long‐term memory without impacting place field representation (Girardeau et al., 2009; Ego‐Stengel & Wilson, 2010; Kovacs et al., 2016), while preventing awake replays impacts working spatial memory and place field stability without globally impacting long‐term memory (Jadhav et al., 2012; Roux et al., 2017). In addition, early reports failed to identify reverse replay in sleep‐based ripples (Lee & Wilson, 2002) and recent studies observe significantly more forwards‐ordered than reverse‐ordered replay in sleep (Wikenheiser & Redish, 2013; Grosmark & Buzsáki, 2016), raising the intriguing possibility that forward replay itself can serve two purposes depending on the behavioral state of the animal (Diekelmann et al., 2011): memory retrieval during the awake state and memory consolidation during sleep (Carr et al., 2011; Lewis & Durrant, 2011). Thus, the phenomenon currently termed “replay” may actually reflect a number of similar but distinct network mechanisms. Furthermore, recent evidence suggests that replay may encode more than simple spatial trajectories, representing both “what” and “where” information simultaneously (Takahashi, 2015), broadening the scope of replay to encompass episodic memory. Finally, it remains unclear if replay reflects conscious, active memory recollection, or whether it instead represents a subconscious and largely automatic mnemonic mechanism. Future work identifying the manner in which hippocampal and extra‐hippocampal areas initiate replay, determine replay content, or respond to replay expression will serve as important steps toward a greater understanding of overall brain function in memory formation and retrieval.

## CONFLICT OF INTEREST STATEMENT

None declared.
